# Aberrant Methylation of *MGMT* Promoter in HNSCC: A Meta-Analysis

**DOI:** 10.1371/journal.pone.0163534

**Published:** 2016-09-22

**Authors:** Fucheng Cai, Xiyue Xiao, Xun Niu, Hao Shi, Yi Zhong

**Affiliations:** 1 Department of Pediatrics, Union Hospital, Tongji Medical College, Huazhong University of Science and Technology, Wuhan, China; 2 Department of Obstetrics and Gynecology, Union Hospital, Tongji Medical College, Huazhong University of Science and Technology, Wuhan, China; 3 Department of Otorhinolaryngology, Union Hospital, Tongji Medical College, Huazhong University of Science and Technology, Wuhan, China; 4 Department of Epidemiology and Biostatistics, and the Ministry of Education Key Lab of Environment and Health, School of Public Health, Tongji Medical College, Huazhong University of Science and Technology, Wuhan, China; Queen Mary University of London, UNITED KINGDOM

## Abstract

**Background:**

O6-methylguanine-DNA methyl-transferase (*MGMT*) gene, a DNA repair gene, plays a critical role in the repair of alkylated DNA adducts that form following exposure to genotoxic agents. *MGMT* is generally expressed in various tumors, and its function is frequently lost because of hypermethylation in the promoter. The promoter methylation of *MGMT* has been extensively investigated in head and neck squamous cell carcinoma (HNSCC). However, the association between the promoter methylation of *MGMT* and HNSCC risk remains inconclusive and inconsistent. Therefore, we performed a meta-analysis to better clarify the association between the promoter methylation of *MGMT* and HNSCC risk.

**Methods:**

A systematical search was conducted in PubMed, Web of Science, EMBASE, and Ovid for studies on the association between *MGMT* promoter methylation and HNSCC. Odds ratio (ORs) and 95% confidence intervals (CI) were calculated to estimate association between *MGMT* promoter methylation and risk of HNSCC. The meta-regression and subgroup analysis were undertaken to explore the potential sources of heterogeneity.

**Results:**

Twenty studies with 1,030 cases and 775 controls were finally included in this study. The frequency of *MGMT* promoter methylation was 46.70% in HNSCC group and 23.23% in the control group. The frequency of *MGMT* promoter methylation in HNSCC group was significantly higher than the control group (OR = 2.83, 95%CI = 2.25–3.56).

**Conclusion:**

This meta-analysis indicates that aberrant methylation of *MGMT* promoter was significantly associated with the risk of HNSCC, and it may be a potential molecular marker for monitoring the disease and may provide new insights to the treatment of HNSCC.

## Introduction

Head and neck squamous cell carcinoma (HNSCC) is the sixth most common malignancy cancer worldwide and about 600,000 new cases each year [1]. Among them, approximately 500,000 HNSCC cases with high malignancy occur each year and the 5-year survival of patients was only 40–50% [2]. And most of HNSCC frequently occurred in the oral cavity, oropharynx, hypopharynx and larynx. At present, tobacco use and alcohol consumption both are well-established risk factors for the development of HNSCC [3]. Moreover, human papillomavirus (HPV) infection has recently been recognized as an independent etiologic factor in the development of HNSCC [4].

Hypermethylation of CpG islands in the promoter region of human genes often resulted in epigenetic inactivation, one of the most frequent events in human tumors. Gene-specific promoter methylation has been increasingly identified as a contributing factor to the development of HNSCC [5,6]. O6-methylguanine-DNAmethyl-transferase (*MGMT*) is a DNA repair gene that plays a crucial role in the mechanism of repair of DNA damage caused by alkylating agents [7]. *MGMT* is widely expressed in various tumors, and its function is frequently lost due to hypermethylation in the promoter. Some studies had found that methylation of MGMT gene promoter was closely related to poor prognosis, metastasis, and recurrence in HNSCC [8–10].

Up to now, many studies have explored the association between aberrant methylation of *MGMT* promoter and HNSCC risk. And most studies aimed to investigate the relationship by comparing the differences in the methylation frequencies of *MGMT* promoter between cancer and non-cancerous. However, the results remain inconclusive and inconsistent. Furthermore, some studies always have a small sample size and have different types of control. Therefore, we conducted a meta-analysis to better clarify the association between *MGMT* promoter methylation and risk of HNSCC.

## Materials and Methods

As described in detail previously [11,12], the meta-analysis was performed according to the latest Preferred Reporting Items for Systematic Reviews and Meta-Analyses (PRISMA).

### Studies Identification

We searched the relevant studies in various online electronic databases (PubMed, Embase, Ovid, and Web of Science). The following search strategy was employed: (oropharyngeal or oral or oropharynx or tonsil or head and neck) and (squamous cell carcinoma or cancer) and (*MGMT* methylation). The search results were updated until May 20, 2016 with restricting to English language.

**Fig 1:**
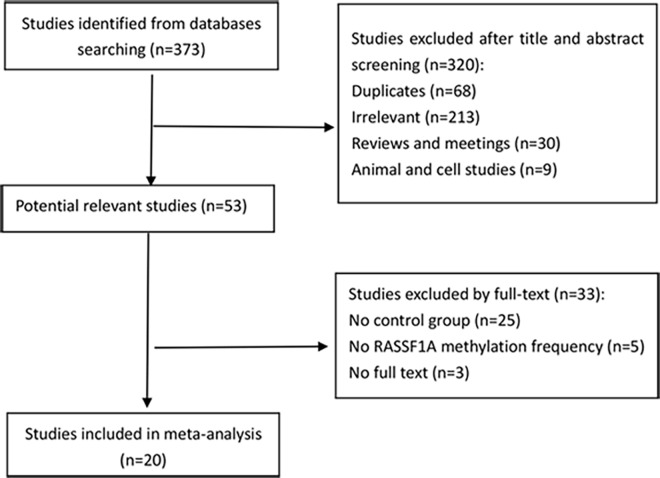
Selection process of studies in the meta-analysis.

The inclusion criteria of the meta-analysis were: (1) articles studying with the association between *MGMT* promoter methylation and HNSCC, (2) case-control study and reporting the *MGMT* promoter methylation frequency in case and control groups, (3) specimens of HNSCC were limited to tissues. Firstly, we read the title and abstract of initial searching articles to assess whether it met the inclusion criteria. Then the potentially relevant articles were evaluated in full-text paper. Finally, a total of 20 articles which contain 1030 cases and 775 controls were included in the meta-analysis. The selection procedure of studies was illustrated in Fig 1.

### Data Extraction and Quality Assessment

Two reviewers (Fucheng Cai and Yi Zhong) independently reviewed the eligible studies. The following information was extracted from the eligible studies: first author’s name, publication year, study population, method for detecting the methylation status, sample type in case and control group, sample sizes (the number of people with *MGMT* methylation and the total people in the case and control groups). All the detailed information extracted from the eligible studies was checked by the third reviewer (Xiyue Xiao).

### Statistical methods

The combined odds ratios (ORs) and its 95% confidence intervals (CIs) were calculated to evaluate the association between *MGMT* promoter methylation and HNSCC risk. The between-study heterogeneity was tested by the *x*^2^-based Cochran Q statistic test and *I*^2^ statistics [13]. The heterogeneity was considered significant (*P*<0.05 for the Q statistic or *I*^2^≥50%) and a random-effects model was used to calculate the pooled ORs. Otherwise, a fixed-effects model was applied to calculate the pooled ORs. Moreover, we performed the meta-regression and subgroup analysis to explore the source of heterogeneity. A sensitivity analysis was executed to investigate the influence of each individual study to the final results of the meta-analysis. The Begg’s funnel plot [14] and Egger’s test [15] were utilized to explore any possible publication bias. For all analyses, the two-sided *P*<0.05 was considered statistically significant. In the Meta package, the default is to add 0.5 to all zero counts when the individual studies have cells with zero counts. All statistical analyses were performed with the Meta package in R (version 3.2.3; http://www.r-project.org/).

## Results

### Study Characteristics

A total of 373 studies were identified by searching the electronic databases.

After eliminating duplicate articles and irrelevant studies (reviews, meeting reviews, and cell lines) by reviewing the titles and abstracts, 53 articles were identified. Finally, 20 studies were included in the meta-analysis by reviewing full-text removing some studies (no control group and no *MGMT* methylation frequency). The search and selection procedures of articles were shown in Fig 1. Among the 20 studies included in our meta-analysis, the control group consisted of HNSCC patients, benign disease patients, and healthy donors and the sample type of control group included tissue, saliva, serum, and buccal cells. The methylation detection methods of the studies included methylation-specific polymerase chain reaction (MSP), real-time quantitative MSP (QMSP), and pyrosequencing. Study characteristics are summarized in Table 1 [16–35].

**Table 1:** Characteristics of studies included in the study.

				Case		Control			Control source	Control sample
Author	Year	Region	Age (case, years)	M	U	M	U	Method		
Onerci Celebi[16]	2016	Turkey	mean = 56.6; range: 40–75	64	18	3	8	Pyrosequencing	H	tissue
Yang[17]	2015	China		27	50	12	50	MSP	A	tissue
Asokan[18]	2014	India		4	6	0	5	MSP	H	tissue
Bhatia[19]	2014	India	mean = 53.0; sd: 12.97	58	18	34	36	MSP	H	tissue
Rettori[20]	2013	Brazil		14	53	2	55	QMSP	H	saliva
Koutsimpelas[21]	2012	Germany	mean = 62.0; range: 45–83	13	10	0	3	MSP	H	tissue
Paluszczak[22]	2011	Poland	mean = 58.3; range: 41–75	22	19	15	26	MSP	A	tissue
Weiss[23]	2011	Germany	mean = 63.7; sd: 11.8	13	39	3	28	MSP	H	tissue
Su[24]	2010	Taiwan	mean = 54.94; range:37–82	7	23	3	27	QMSP	A	tissue
						0	12		H	buccal cell
Kordi[25]	2010	Iran	mean = 54.14	56	20	31	26	MSP	H	tissue
Steinmann[26]	2009	Germany	mean = 57.0; range: 41–77	29	25	7	16	MSP	A	tissue
De Schutter[27]	2009	Belgium		17	23	0	5	MSP	H	tissue
Righini[28]	2007	France	median = 57.0; range: 33–7	20	70	0	30	MSP	A	tissue
						0	30		H	saliva
						13	47		A	saliva
Martone[29]	2007	Italy	mean = 60.9; range: 27–89	10	10	5	6	MSP	A	tissue
Kato[30]	2006	Japan		27	24	0	18	MSP	H	tissue
						9	13		A	tissue
Maruya[31]	2004	USA	mean = 58.3; range: 31–81	10	22	7	25	MSP	A	tissue
						1	5	MSP	H	tissue
Kulkarni[32]	2004	India	mean = 50.0; range:25–71	31	29	16	44	MSP	A	tissue
						0	20	MSP	H	buccal cell
Viswanathan[33]	2003	India		21	30	0	25	MSP	A	tissue
Rosas[34]	2001	USA		7	23	4	3	MSP	A	saliva
						1	29		H	saliva
Sanchez[35]	2000	USA		31	64	14	15	MSP	A	serum

M: MGMT promoter methylated, U: MGMT promoter unmethylated

A: Autologous, H: Heterogeneous

sd: standard deviation

### Meta-analysis

**Fig 2:**
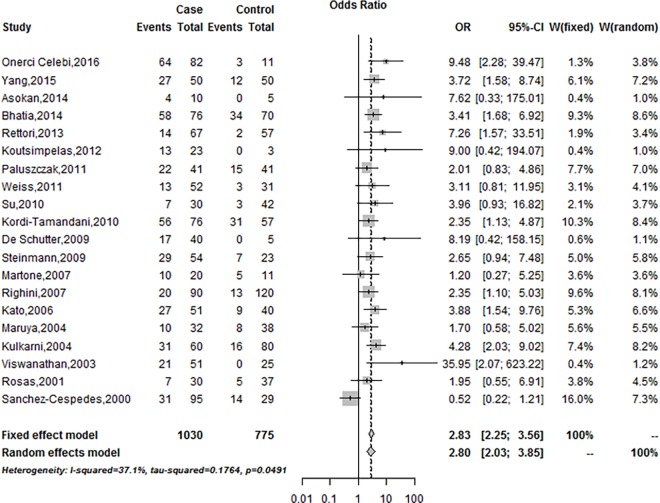
Forest plot of *MGMT* promoter methylation associated with HNSCC risk under the fixed-effects model.

The combining result of the association of *MGMT* promoter methylation with HNSCC risk was shown in Fig 2. The fixed-effects model was employed due to the significant heterogeneity among the included studies (*I*^*2*^ = 37.1%, *P* = 0.05). *MGMT* promoter methylation frequency was significantly associated with an increased HNSCC risk based on the fixed effects model (Summary OR was 2.83, 95%CI = 2.25–3.56) (Fig 2).

### Meta-regression and Subgroup Analysis

Although the heterogeneity among the studies was found no significant (*I*^*2*^ = 37.1%, *P* = 0.05), we also conducted the meta-regression to find the potential sources of heterogeneity. The results of meta-regression showed that the potential source of the heterogeneity was only found in the control source (*P* = 0.02) (Table 2). We also performed the subgroup analysis to further evaluate the source of the heterogeneity according to race, method, control source, control sample type, and case sample size.

**Table 2:** Meta-regression analysis.

		95%CI		
Heterogeneity sources	Coefficient	Lower	Upper	*P*
Publication year	0.011	-0.096	0.118	0.839
Population	-0.297	-1.106	0.511	0.471
Method	0.538	-0.702	1.777	0.395
Case sample size	0.078	-0.719	0.874	0.849
Control source	0.982	0.131	1.832	0.024
Control sample	0.450	-0.610	1.510	0.406

In the race subgroup analysis, the OR in Asians group was 3.82 (95%CI = 2.75–5.30) under the fixed-effects model, and 2.28 (95%CI = 1.40–3.71) in Caucasians group under the random-effects model. Onerci Celebi [16] used pyrosequencing to detect *MGMT* promoter methylation and we have put the study classified as QMSP group in the methylation detection method. The OR for was 2.62 (95%CI = 2.07–3.33) in the MSP group under, and 6.48 (95%CI = 2.76–15.18) in the QMSP group under the fixed-effects model. With the control source, the OR in autologous group was 1.78 (95%CI = 1.12–2.81) under the random-effects model, and 5.18 (95%CI = 3.56–7.53) in the heterogeneous group under the fixed-effects model. In the control sample type group, we classified the studies into non-tissue group (control sample type: serum, saliva and buccal cell) and tissue group (control sample type: tissue).The OR for was 2.61 (95%CI = 0.81–8.41) in the non-tissue group, and 3.14 (95%CI = 2.03–4.86) in the tissue group under the random-effects model. The OR for was 2.62 (95%CI = 1.72–3.99) in case sample size < = 50 group under the fixed-effects model, and 3.05 (95%CI = 1.89–4.92) in case sample size >50 under the random-effects model. The results of subgroup analysis were summarized in Table 3.

**Table 3:** Summary of the subgroup analysis.

	Case		Control		M-H pooled OR^ƛ^	D+L pooled OR^ǂ^	Heterogeneity		
Group	M+	N	M+	N	OR (95%CI)	OR (95%CI)	*I*^*2*^ (%)	*P*	τ^2^
Total	481	1030	180	775	2.83 (2.25–3.56)	2.80 (2.03–3.85)	37.1	0.05	0.18
Population subgroup									
Asians	231	404	105	369	3.82 (2.75–5.30)	3.57 (2.56–4.99)	0	0.73	0
Caucasians	250	626	75	406	2.13 (1.55–2.94)	2.28 (1.40–3.71)	46	0.04	0.31
Case sample size									
≤50	117	276	48	232	2.62 (1.72–3.99)	2.52 (1.64–3.88)	0	0.78	0
>50	364	754	132	543	2.92 (2.22–3.84)	3.05 (1.89–4.92)	60.4	<0.01	0.36
Control source									
Autologous	242	604	105	420	1.93 (1.45–2.56)	1.78 (1.12–2.81)	54.1	0.01	0.33
Heterogeneous	341	719	75	355	5.18 (3.56–7.53)	4.31 (2.88–6.46)	2.2	0.43	0.01
Control sample type^$^									
Tissue	429	838	146	530	2.91 (2.30–3.68)	3.14 (2.03–4.86)	58.2	<0.01	0.52
Non-tissue	110	372	34	245	2.78 (1.56–4.96)	2.61 (0.81–8.41)	59.6	0.03	1.16
Method									
MSP	396	851	172	665	2.62 (2.07–3.33)	2.52 (1.82–3.50)	35.9	0.07	0.15
QMSP	85	179	8	110	6.48 (2.76–15.18)	6.48 (2.78–15.11)	0	0.69	0

ƛ: the fixed-effects model

ǂ: the random-effects model

$: Non-tissue: serum, saliva and buccal cell

### Sensitivity Analysis

**Fig 3:**
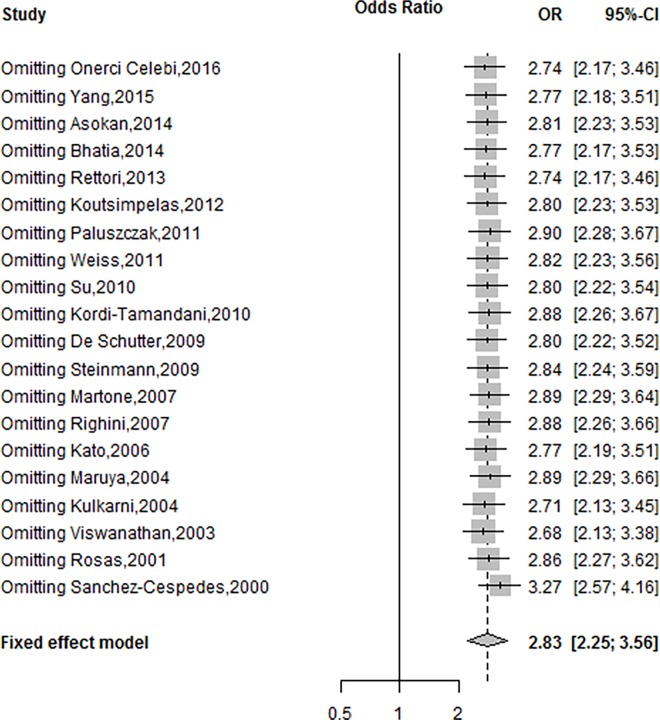
Sensitivity analysis of included studies under the fixed-effects model.

To evaluate the effects of each individual study on the overall effect, the sensitivity analysis was performed. The overall OR was changed from 2.68 (95%CI, 2.13–3.38) to 3.27 (95%CI, 2.57–4.16) under the fixed effects model by omitting each single study, which demonstrates that the pooled OR between the *MGMT* promoter methylation and risk of HNSCC was reliable and stable (Fig 3).

### Publication Bias

**Fig 4:**
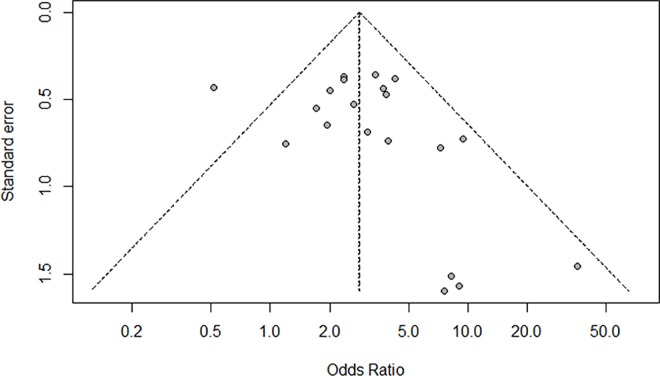
Funnel plot for assessment of publication bias in the meta-analysis.

The Begg’s funnel plot and Egger’s test were employed to estimate the publication bias of the included studies. The Begg’s funnel plot of the pooled analysis in Fig 4 was quite symmetric and no publication bias was detected by Egger’s test (*P* = 0.31).

## Discussion

Epigenetic inactivation of the genes is common in human tumors. Hypermethylation of gene promoter is one of the important mechanisms for inactivation of tumor-suppressor genes involving apoptosis, DNA-repair, and cell cycle control [36]. The *MGMT* is a DNA repair gene, and the promoter methylation of *MGMT* plays an important role in the carcinogenic process and progression of cancer [37]. The protein *MGMT* also known as AGT (O6-alkylguanine transferase), is well known to play a crucial role in repairing O6-alkylguanine in DNA, a major premutagenic lesion produced by environmental and therapeutic alkylating agents [38]. Methylation of *MGMT* gene promoter can diminish *MGMT* protein expression in tumor tissues of various types of cancers, including lung cancer, gastric cancer, colorectal cancer, and breast cancer [39–43].

To our knowledge, this is the first meta-analysis on evaluating the association between *MGMT* promoter methylation and the risk of HNSCC. In this study, we found that 20 studies including 1030 cases and 775 controls were competent for inclusion criteria. The frequency of *MGMT* promoter methylation in tumor was 46.70% and 23.23% in control group. The result of the meta-analysis displayed that the methylation of *MGMT* promoter had an increased risk of HNSCC (OR = 2.83; 95%CI = 2.25–3.56).

Subgroup analysis by the methylation detection method showed the OR was 2.62 (95%CI = 2.07–3.33) in the MSP group and 6.48 (95%CI = 2.76–15.18) in the QMSP group under the fixed-effects model. In fact, QMSP is reported to be more specific and more sensitive, and able to detect much smaller magnitude (1/1000 methylated alleles) [44,45]. In contrast, the conventional MSP can only detect high concentrations of promoter methylation.

With the control style, the OR was 5.18 (95%CI = 3.56–7.53) in the heterogeneous control subgroup under the fixed-effects model and 1.78 (95%CI = 1.12–2.81) in autologous tissues subgroup under the random-effects model. The result suggested that the incidence of *MGMT* methylation in autologous control was higher than that in heterogeneous control. The result of the subgroup analysis is consistent with most previous studies [24,30,32]. In the control sample type subgroup, the OR was 3.14 (95%CI = 2.03–4.86) in the tissue group and 2.61 (95%CI = 0.81–8.41) in the non-tissue group under the fixed-effects model. In the subgroup analysis by sample size and race, significant associations were observed for all subgroups.

However, the present study had also several potential limitations. First, the search strategy was restricted to articles published in English language in this study. And then, some studies potentially suitable for inclusion that were published in other languages may be not included. Thus, some publication bias may exist. Second, we only study the association between *MGMT* promoter methylation and HNSCC in this study. We did not further investigate the association between *MGMT* promoter methylation and demographic (age and gender) and disease characteristics (stage, metastasis, and relapse) of HNSCC.

In conclusion, we found that hypermethylation of *MGMT* promoter was associated with an increased risk of HNSCC. The findings suggested that the promoter methylation of MGMT gene may play an important role in the carcinogenic process of HNSCC, and it may be a promising molecular marker for monitoring the disease and may provide new insights to the treatment of HNSCC. However, more studies with larger sample size must be performed to acquire a more precise and representative result.

## Supporting Information

S1 Checklist PRISMA Checklist. (DOC)

S2 Checklist *PLOS ONE* Clinical Studies Checklist. (DOCX)

S3 Checklist Meta-analysis on Genetic Association Studies Checklist. (DOCX)
